# Leptomeningeal Seeding of Pituitary Adenoma in a Young Lady With Neglected Gigantism: An Extremely Rare Case Report

**DOI:** 10.1002/ccr3.70342

**Published:** 2025-04-04

**Authors:** Sadegh Bagherzadeh, Milad Shafizadeh, Seyed Mohammad Tavangar, Alireza Khoshnevisan

**Affiliations:** ^1^ Department of Neurosurgery Shariati Hospital, Tehran University of Medical Sciences Tehran Iran; ^2^ Sports Medicine Research Center Neuroscience Institute, Tehran University of Medical Sciences Tehran Iran; ^3^ Department of Pathology Shariati Hospital, Tehran University of Medical Sciences Tehran Iran

**Keywords:** acromegaly, gigantism, leptomeningeal, pituitary adenoma, somatotroph

## Abstract

Pituitary neuroendocrine tumors can sometimes present with leptomeningeal seeding at their initial diagnosis, emphasizing the need for thorough evaluation in cases of leptomeningeal involvement. Additionally, it is crucial for general physicians to inquire about a patient's menstrual status, particularly in low socioeconomic conditions, as this information can provide valuable insights into the hypothalamic–pituitary axis function.

## Graphical abstract

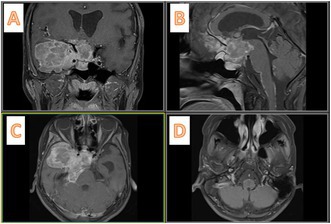

## 1 Introduction

Gigantism results from an excess of growth hormone that begins before puberty. Symptoms typically include large hands and feet, thick fingers and toes, a prominent jaw and forehead, and coarse facial features [1]. Approximately 95% of cases of acromegaly and gigantism are attributed to a growth hormone‐secreting adenoma in the pituitary gland. In addition, secretion of growth hormone‐releasing hormone (GHRH) from a hypothalamic adenoma or ectopic GHRH production from neuroendocrine tumors in the lungs or pancreas can also contribute to acromegaly [2]. Genetic syndromes associated with hypersecretion of growth hormone include multiple endocrine neoplasia type 1 (MEN‐1), neurofibromatosis, Carney complex, and McCune‐Albright syndrome [3]. In this case report, we will report an extremely rare primary leptomeningeal seeding in a young lady with neglected gigantism.

## 2 Case History/Examination

### 2.1 History

A 21‐year‐old female of Afghan descent was referred to our pituitary clinic with a primary complaint of progressively worsening eyesight over the past 10 years. The patient had no significant medical or surgical history and was not on any medications. She reported experiencing mild headaches, described as tension‐type headaches. Additionally, she is not married and has never had menstruation.

### 2.2 Physical Examination

**FIGURE 1:**
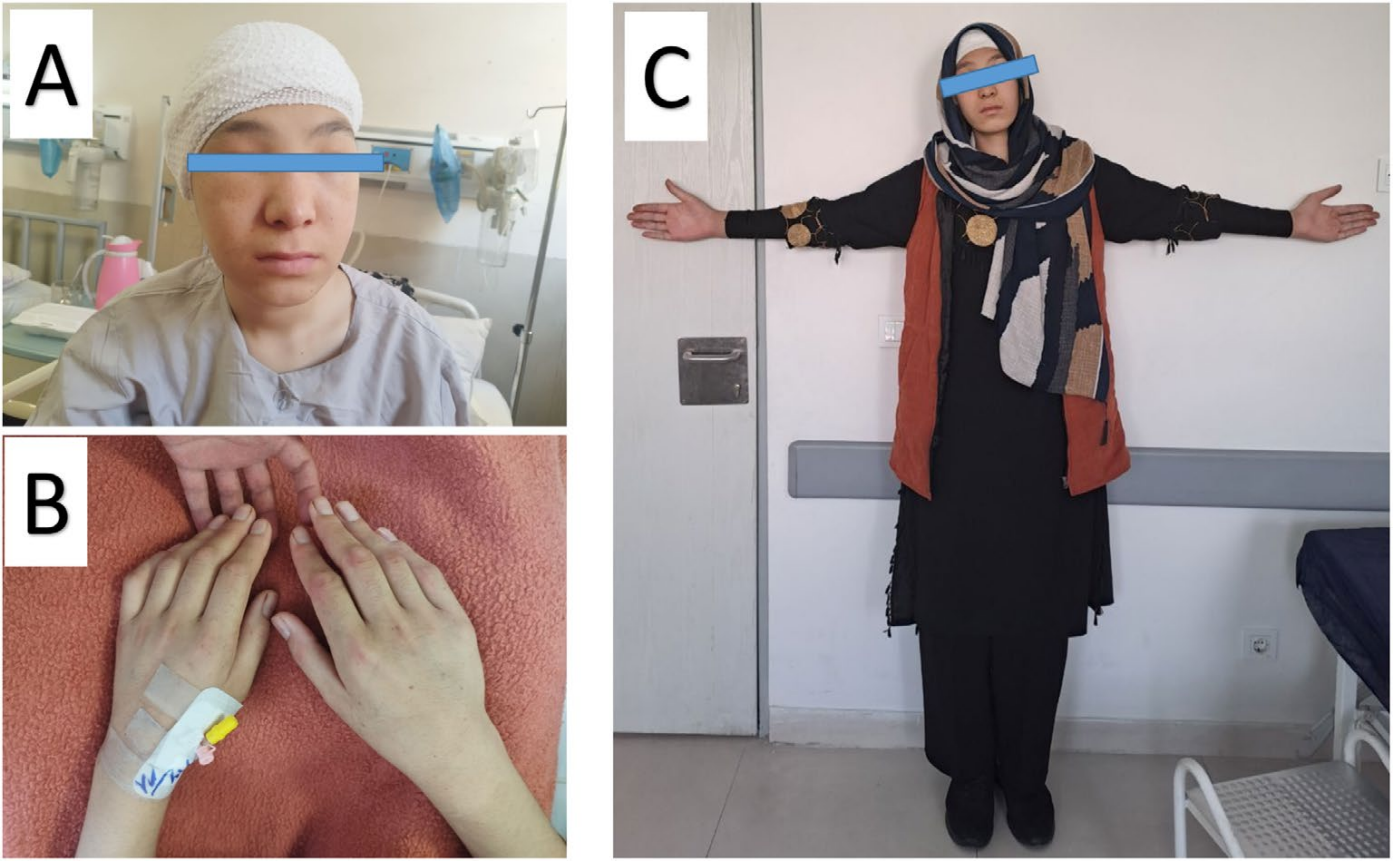
Clinical features of gigantism in our patient. (A) The patient has a puffy face due to hypothyroidism. (B) Comparison between the size of the examiner's hand and the patient's hand. (C) The patient was taller than other members of the family.

She appeared slightly lethargic, with a low tone of voice, apathetic behavior, and a depressed attitude. Visually, she had periorbital edema, a puffy face, and notable features of acromegaly, including large hands. Her height was 196 cm (Figure 1). During the general medical examination, her blood pressure was recorded at 100/75 mmHg, her heart rate was 67 beats per minute, and her body temperature was 36.4°C. Lung and heart examinations showed no abnormal findings. Additionally, she had non‐pitting edema in both legs, rated as 1+ in severity.

The neurological examination revealed that the sensory assessment was normal, aside from pinprick hypesthesia on the right side of her face. The motor evaluation indicated normal muscle strength but a significant decrease in muscle tone. The reflex assessment showed generalized hyporeflexia in deep tendon reflexes (1+). Eye movements were normal in both eyes, and other cranial nerve evaluations were within normal limits. During the cranial nerve examination, visual acuity was evaluated, with the right eye only able to perceive hand motion, while the left eye could count fingers at a distance of 30 cm. Due to the limited visual acuity, the visual field examination was deferred. Fundoscopic examination showed right optic nerve atrophy, and the left optic nerve had Frisen Grade I edema. The pupillary exam revealed a relative afferent pupillary defect in the right eye.

## 3 Methods (Differential Diagnosis, Investigations, and Treatment)

### 3.1 Diagnostic Assessment

**FIGURE 2:**
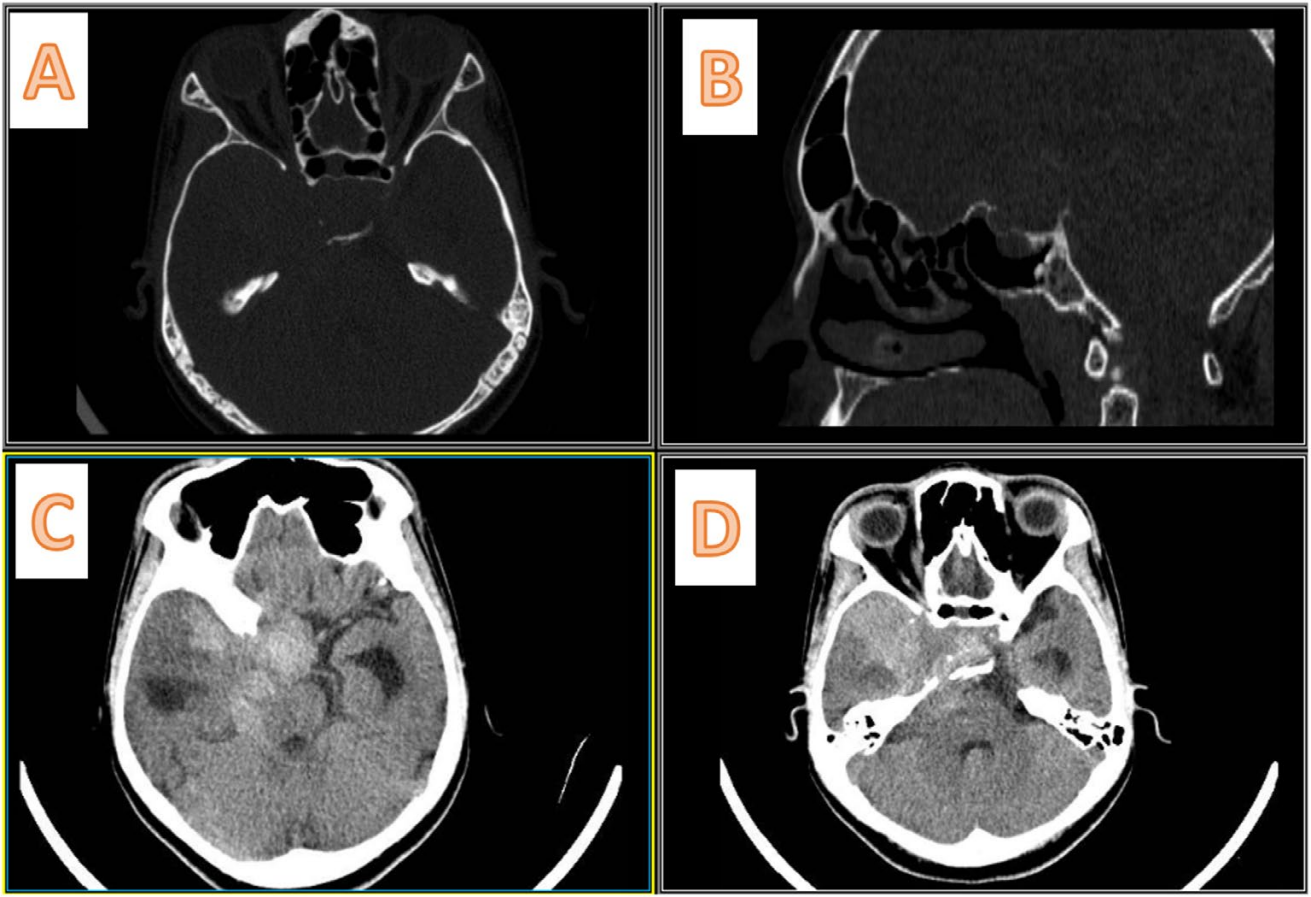
Preoperative non‐contrast enhanced brain CT scan. (A, B) The bone window; there is a widening of the sella turcica in A and Erosion of the sella floor in sagittal view in (B). (C, D) The parenchymal view of the brain; there is a heterogeneous hyperdense lesion in the sella turcica with extension to the right middle fossa and posterior fossa through the tentorium cerebelli. There are enlarged temporal horns of the lateral ventricles bilaterally, which indicate hydrocephalus.

**FIGURE 3:**
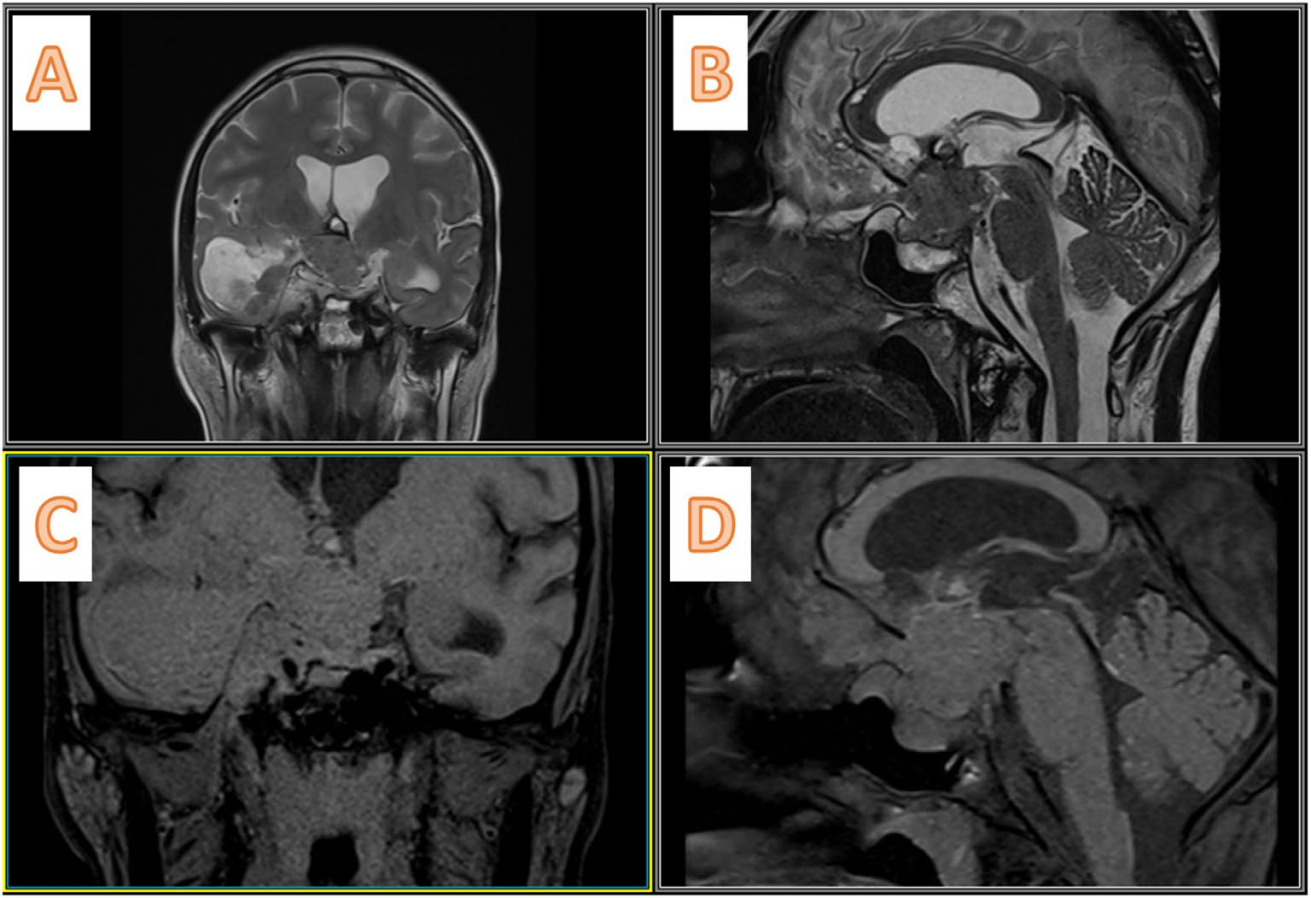
Preoperative non‐contrast enhanced MRI. (A) T2 weighted coronal image depicts the solid cystic lesion extending from sella turcica to the right middle fossa and abutting the Meckel's cave. (B) T2 weighted sagittal image shows iso to hyperintense mass within the sella cavity with extension to the third ventricle floor and compressive effect on the foramen of the Monro, causing biventricular hydrocephalus. (C, D) T1 weighted coronal and sagittal images indicate iso to hyperintense lesion.

**FIGURE 4:**
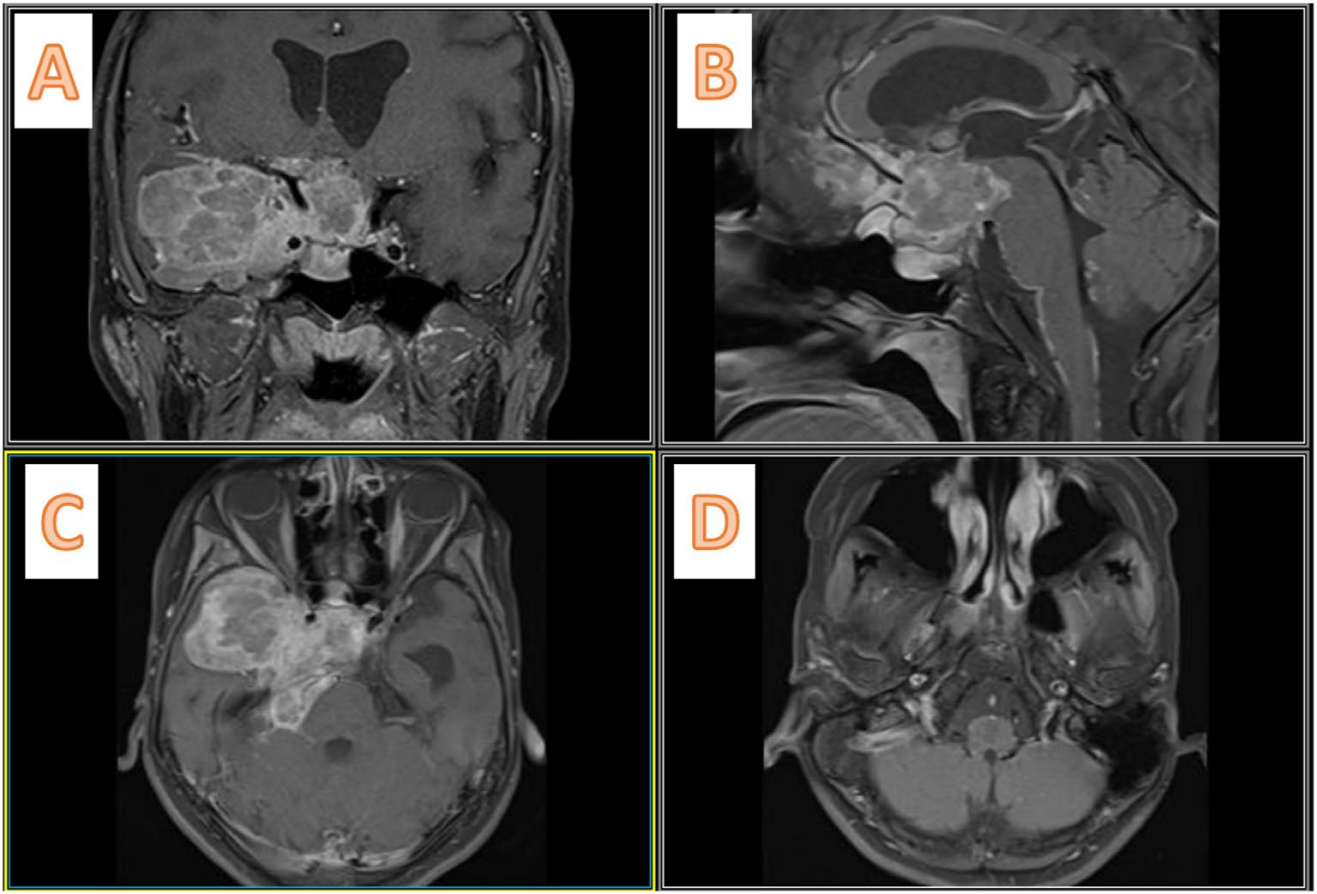
Preoperative Gadolinium‐enhanced MRI. (A) Coronal cut indicates heterogeneously enhancing lesion in sella turcica with Knosp Grade 4 on the right side and encasement of the carotid bifurcation. (B) Sagittal cut shows fine nodular enhancement over the leptomeninges of the brainstem, along the anterior cerebral artery, and over the medial surface of the frontal lobe. (C, D) The lesion extends through the tentorium cerebelli to the posterior fossa; as depicted in (D), there is leptomeningeal enhancement over the vestibulocochlear complex.

She came with a non‐enhanced CT scan of the brain (Figure 2), which showed a mixed‐density mass in the right middle fossa and sella region, extending to the posterior fossa through the incisura. The sella cavity was enlarged and showed bony destruction. We performed a gadolinium‐enhanced MRI of the brain, which demonstrated mixed hypointensity on T1 and mixed hyperintensity on T2 (Figure 3), along with heterogeneous enhancement. A cystic component was noted in the right middle fossa, as well as a posterior fossa component. Following the gadolinium injection, we observed leptomeningeal enhancement with seeding between the frontal lobes, over the brainstem, around the VII/VIII cranial nerves, and near the jugular foramen (Figure 4).

Our initial biochemistry results were largely within normal limits; however, the hormonal laboratory findings indicated several abnormalities. The 8 AM serum cortisol was decreased at 1.9 (with a normal lower limit of 5), and T4 was also decreased at 4.2 (normal lower limit: 4.8). Prolactin levels were found to be elevated at 63 (normal upper limit: 24), while growth hormone was greater than 50 (normal range: up to 8). Additionally, IGF‐1 was notably elevated at 435 (normal range: up to 270). All other hormonal tests remained within normal limits.

### 3.2 Therapeutic Interventions

The primary differential diagnosis was a growth hormone‐producing pituitary adenoma. Due to low T4 and cortisol levels, treatment was initiated with levothyroxine at 100 μg per day and hydrocortisone at 100 mg three times daily. Three weeks later, the patient underwent a right pterional craniotomy and transcranial subtotal resection of the tumor. During surgery, the tumor appeared yellowish‐gray with a soft consistency, allowing for easy suction, and there was no excessive bleeding, resulting in an estimated blood loss of 400 cc over 200 min.

**FIGURE 5:**
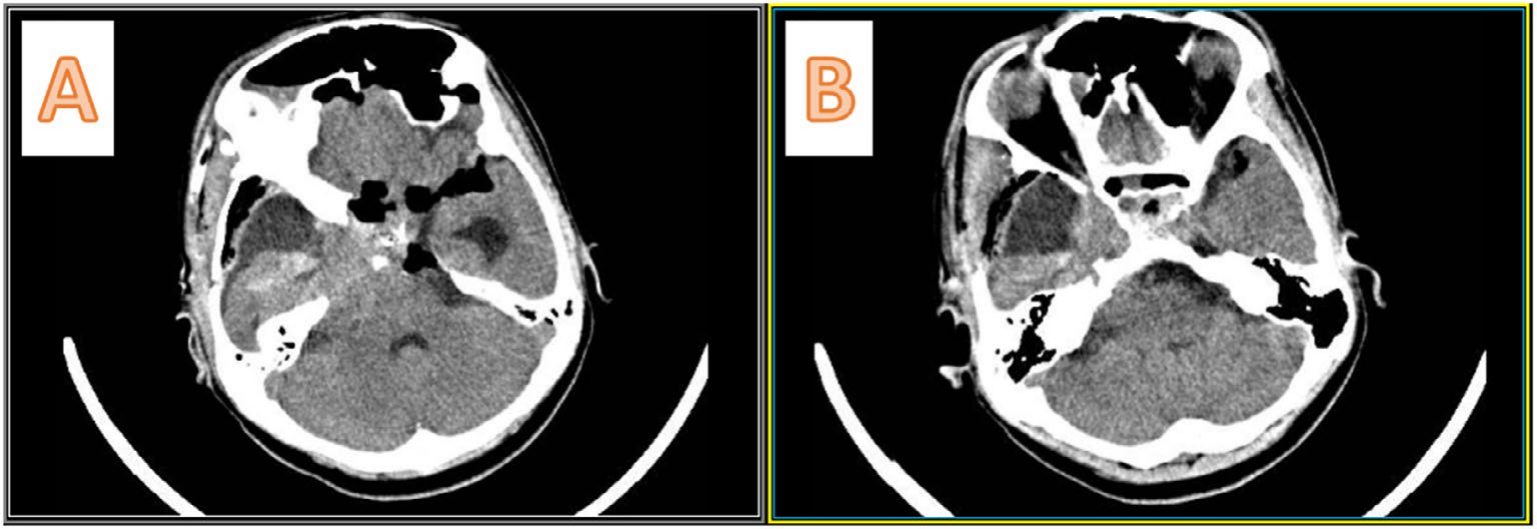
Postoperative non‐contract enhanced brain CT scan. (A, B) Subtotal resection of the mass with complete removal of the middle fossa and sella components and remnant of the posterior fossa component.

Figure 5 shows the postoperative CT scan. She was extubated 2 h later in the ICU. Although there was no improvement in the right eye's visual acuity, the left eye showed slight improvement, with the patient able to count fingers at about 1 m. The patient did not exhibit signs of diabetes insipidus. On the second postoperative day, laboratory tests revealed decreased growth hormone levels to 30 and IGF‐1 at 327. The patient was discharged on the fifth day from the hospital with a prescription for levothyroxine at 100 mcg per day and hydrocortisone at 20 mg twice daily.

## 4 Results (Outcome and Follow‐Up)

### 4.1 Pathology Examination

**FIGURE 6:**
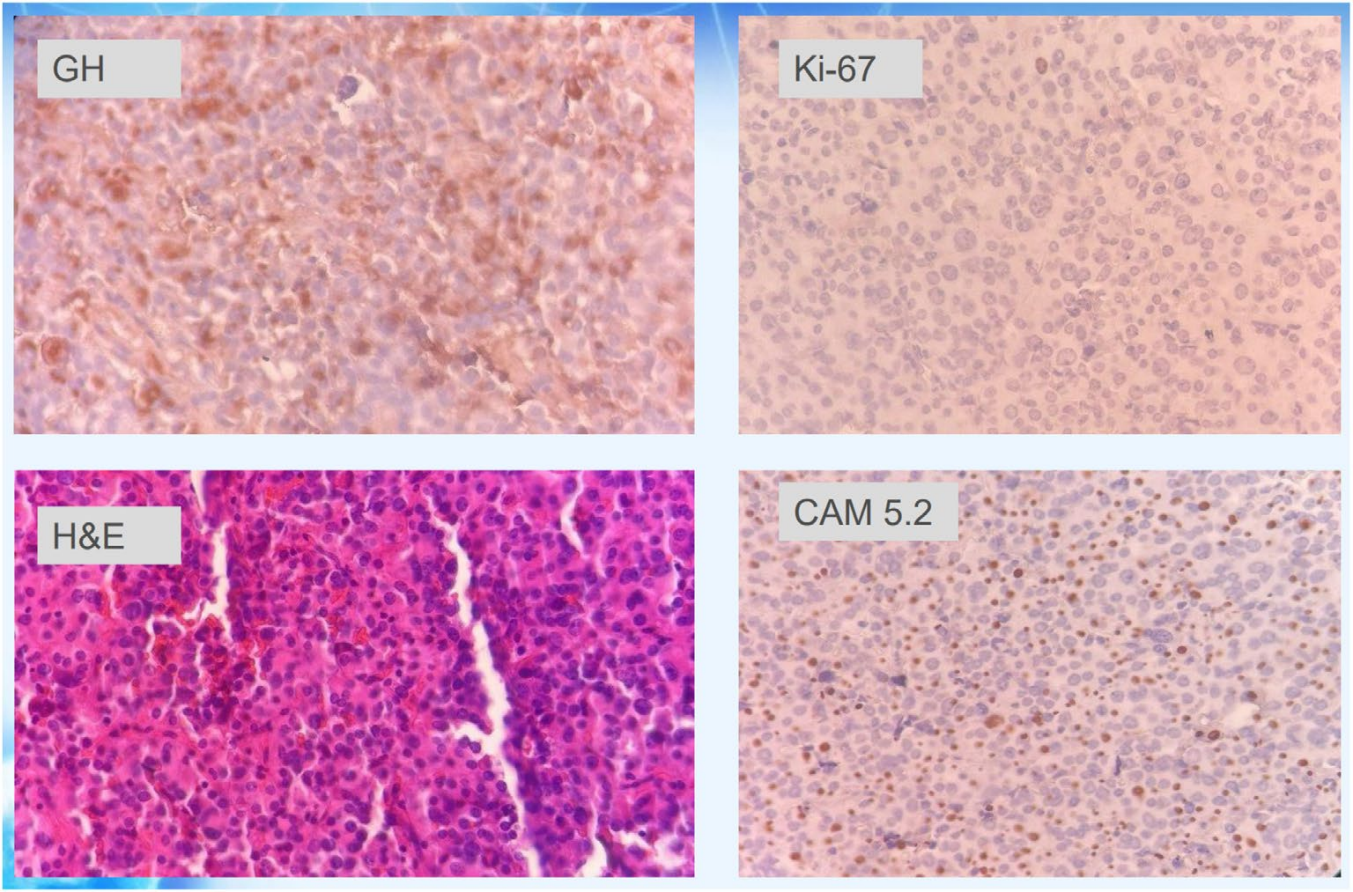
Results of Immunohistochemical staining, which indicates the sparsely granulated growth hormone‐producing pituitary neuroendocrine tumor.

Pathological examination revealed sheets and nests of large cells with eosinophilic, granular cytoplasm and a central nucleus containing prominent nucleoli, as shown in Figure 6. The specimen was positive for immunohistochemistry (IHC) markers CAM 5.2, chromogranin, synaptophysin, and growth hormone (GH), indicating a somatotroph pituitary neuroendocrine tumor (Pit‐NET). P53 was negative, while Ki‐67 and MIB‐1 showed positivity in less than 1% of tumor cells. Based on the pattern of GH IHC, the pathological diagnosis was determined to be sparsely granulated somatotroph Pit‐NET.

### 4.2 Outcome and Follow‐Up

The patient visited 3 weeks post‐discharge, during which time her symptoms of hypothyroidism and hypocortisolism showed significant resolution. Due to the identified pattern of leptomeningeal metastasis, as well as findings in the pathological report indicating sparsely granulated somatotroph adenoma, the patient has been referred to a radio oncologist for the initiation of chemoradiotherapy. The patient underwent chemotherapy with temozolomide, and in the third‐month follow‐up, her endocrinological tests were within normal limits, except her growth hormone level was 20, and IGF1 was 253 at the third‐month follow‐up.

## 5 Discussion

The identification of metastatic PitNETs predominantly relies on the detection of craniospinal and/or systemic metastases, especially in cases where malignant histological characteristics are not present [4]. In cases of metastatic PitNETs, the most commonly observed types are corticotroph and lactotroph tumors. This highlights their prevalence in metastatic situations related to pituitary tumors [5, 6].

Tanaka et al. [7] reported a 70% incidence of metastasis following surgical intervention in primary PitNETs. Furthermore, there have been documented cases of PitNETs metastasizing along the surgical trajectory [7, 8]. However, to our knowledge and after thoroughly searching the literature, we found no case report of leptomeningeal disease as the first presentation of the PitNETs.

While a single biomarker may not be sufficient to predict tumor behavior, studies have demonstrated that at least one marker can be useful in most cases involving metastatic PitNETs. Notably, a Ki‐67 index exceeding 3% is the most commonly observed positive marker in metastatic PitNETs. Additionally, P53 positivity and a mitotic count of more than two mitoses per 10 high‐power fields (HPFs) are frequently documented features in these tumors [9, 10].

Unfortunately, there is little information about the pathophysiology of this phenomenon. In previous case reports, secondary seeding due to surgical manipulation and the opening of the tumor capsule has been proposed as mechanisms. However, due to the rarity of this phenomenon, there may be a cellular and genetic explanation for this behavior of PitNETs. We hypothesize that the nature of the patient's PitNET (which is sparsely granulated and GH‐producing), considered a poor prognostic type, along with the long‐standing presence of the tumor due to neglect, may contribute to the unusual presentation of this tumor in our patient.

The European guidelines for managing aggressive PitNETs [9] recommend that surgery should be conducted by a neurosurgeon who has extensive experience in pituitary surgery. For aggressive pituitary tumors and pituitary carcinomas, temozolomide (TMZ) monotherapy is recommended as the first‐line chemotherapy. According to published literature, the estimated overall response rate to TMZ in patients is 47% (with a 95% confidence interval of 36%–58%) [11]. Additionally, adjuvant radiotherapy should be considered in cases where there is a clinically significant invasive tumor remnant.

Our case report has several points for practitioners in the field of endocrinology and neurosurgery. Firstly, we should consider PitNET as a rare differential diagnosis in cases of leptomeningeal seeding; secondly, asking about menstruation status should be considered in general physician visits, especially in low socioeconomic conditions. Thirdly, when we decide to proceed with the observation of PitNET or after surgical removal of the adenoma, we should be aware of this rare complication.

## 6 Conclusion

We presented a rare case of primary leptomeningeal seeding in a young female with a GH‐producing PitNET. The patient was treated with surgical excision and chemotherapy. We should consider the PitNET as a differential diagnosis of leptomeningeal seedings.

## Author Contributions

**Sadegh Bagherzadeh:** investigation, software, visualization, writing – original draft. **Milad Shafizadeh:** writing – review and editing. **Seyed Mohammad Tavangar:** investigation. **Alireza Khoshnevisan:** supervision, writing – review and editing.

## Ethics Statement

Our institution's ethical committee waived ethical approval for this case report because it was considered part of the usual patient care.

## Consent

The patient gave verbal and informed written consent to the use of his clinical data and images for publication in this case report; no identification of the patient's identity is present either in the manuscript or in the images.

## Conflicts of Interest

The authors declare no conflicts of interest.

## Data Availability

The data that support the findings of this study are available on request from the corresponding author. The data are not publicly available due to privacy or ethical restrictions.
